# Engineering Anisotropic Mechanical Properties in Large‐Scale Fabricated Cartilage Constructs Using Microfiber Reinforcement

**DOI:** 10.1002/adhm.202501014

**Published:** 2025-06-08

**Authors:** Lennard Spauwen, Alba Pueyo Moliner, Patrick van Veenendaal, Roel Custers, Jos Malda, Mylène de Ruijter

**Affiliations:** ^1^ Department of Orthopedics University Medical Center Utrecht Heidelberglaan 100 Utrecht 3584 CX The Netherlands; ^2^ Regenerative Medicine Center Utrecht Uppsalalaan 8 Utrecht 3584 CT The Netherlands; ^3^ University of Applied Sciences Utrecht Padualaan 99 Utrecht 3584 CH The Netherlands; ^4^ Department of Equine Sciences Faculty of Veterinary Sciences Utrecht University Yalelaan 108 Utrecht 3584 CM The Netherlands

**Keywords:** anisotropic scaffolds, cartilage, melt electrowriting, regenerative medicine

## Abstract

Despite the growing prevalence of cartilage damage in the knee joint, effective regenerative treatments remain limited. One of the current challenges is the accurate matching of the local mechanical properties of the tissue, which vary throughout the articular joint surface. This study investigates the fabrication of cartilage constructs with anisotropic mechanical properties. Specifically, it aims to develop composite constructs by reinforcing gelatin‐methacryloyl (gelMA) hydrogels with melt electrowritten (MEW) fibers arranged to mimic the surface anisotropic mechanical properties of the native articular cartilage. Large‐size anisotropic MEW scaffolds are successfully generated, after which they are embedded in the hydrogel, yielding stable constructs. Local mechanical properties can be tailored by varying the fiber spacing while providing a suitable environment for Articular Cartilage Chondroprogenitor cells (ACPCs) to deposit a cartilage‐like matrix. Importantly, unlike reinforcement with fibers generated with fused deposition modeling (FDM), reinforcement with MEW avoided stress shielding, thereby facilitating cell response. This highlights the potential of these reinforced constructs to further match local tissue characteristics and provide a durable solution for the restoration of larger cartilage defects.

## Graphical abstract

This study presents the development of large‐scale (15 cm^2^) cartilage constructs with anisotropic mechanical properties using melt electrowritten (MEW) microfiber reinforced cell‐laden gelatin methacryloyl (gelMA). These constructs mimic the mechanical behavior of native cartilage, providing a suitable environment for Articular Cartilage Chondroprogenitor cells (ACPCs) to deposit cartilage‐like matrix, offering a promising solution for repairing large cartilage defects.

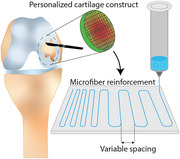

## 1 Introduction

Articular cartilage (AC) is a specialized connective tissue that covers the surface of articulating bones, and its thickness ranges from 0.5 to 4 mm, with an average of 2 mm in the femoral condyle (the end of the thighbone that connects with the tibia to form the knee joint).^[^ ^1^, ^2^, ^3^, ^4^ ^]^ Patients with cartilage damage experience significant pain and mobility issues as AC plays a vital role in the smooth movement of our joints, providing a near‐frictionless surface and efficiently transferring loads from the joint surface to the underlying bone.^[^ ^5^ ^]^ Healthy AC has an extremely low coefficient of friction (0.001), yet can endure pressures up to 100 bar, several times the body weight during dynamic movements.^[^ ^6^ ^]^ Localized cartilage damage – typically affecting isolated areas – can lead to early onset osteoarthritis (OA) when left untreated, increasing pain in patients.^[^ ^1^, ^7^, ^8^ ^]^ A total knee arthroplasty (TKA) is an effective treatment for OA, yielding favorable patient outcomes.^[^ ^9^ ^]^ However, for younger patients (specifically men in their early 50s), the revision rate is significantly higher, as the durability is limited and is prone to wear of the polyethylene friction surface, which also causes osteolysis.^[^ ^9^, ^10^, ^11^ ^]^ Small osteochondral defects, not classified as full‐scale OA, have several biological treatment options, though each comes with limitations. Osteochondral allografts result in good outcomes but are only available in specific countries and dependent on donor availability.^[^ ^12^ ^]^ Ostechondral autografts result in donor side morbidity, especially for larger implants.^[^ ^13^, ^14^ ^]^ Regenerative, biodegradable treatments remain a challenge due to the need for personalization in biomechanics, size, and shape, and print‐on‐demand availability.

The characteristics of AC are derived from its extracellular matrix (ECM), mainly composed of negatively charged proteoglycans and collagen type II. Aggrecan, the most prevalent proteoglycan, is crucial for water retention, resulting in up to 95% water content. The mechanical strength of AC comes from the structural support from collagen fibers and their interaction with the proteoglycan network.^[^ ^3^, ^15^ ^]^ The collagen network is oriented in an arcade structure, also referred to as the “Benninghoff” arcades.^[^ ^15^, ^16^ ^]^ This arcade structure restricts proteoglycan‐associated water swelling and allows the tissue to withstand high compressive loads. The loading of AC is associated with the local collagen fiber density and orientation in the superficial layer, resulting in greater alignment in loaded regions when compared to non‐loaded regions.^[^ ^17^ ^]^ As a result, the AC surface exhibits not only anisotropic mechanical properties not only through its depth, but also across different surface locations.

Biofabrication holds promise for accurately depositing hydrogel‐based bioinks, which can further capture the complexity of tissues, potentially creating constructs that can restore the native mechanical function of AC.^[^ ^18^, ^19^, ^20^, ^21^ ^]^ Various hydrogel‐based materials have already been developed and shown to provide a native‐like environment for cartilage cells.^[^ ^22^, ^23^, ^24^, ^25^ ^]^ However, these soft hydrogels do not reflect the mechanical support of native tissue, as the initial mechanical properties are predominantly determined by the crosslink density.^[^ ^26^ ^]^ Efforts to develop high‐strength hydrogels continue to encounter challenges related to complex preparation methods, control of mechanical properties, toxicity levels, and deposition of cartilaginous neo‐tissue.^[^ ^27^, ^28^, ^29^ ^]^ Due to the lack of mechanical stiffness in the hydrogels, embedded chondrocytes do not perceive adequate mechanical stimulation and tend to lose their phenotype, resulting in a fibroblastic appearance and dedifferentiation.^[^ ^30^, ^31^ ^]^ Fused deposition modelling (FDM) with thermoplastic polymers has been used to reinforce these soft, cell‐laden hydrogels, obtaining hybrid structures.^[^ ^32^ ^]^ By altering the shape and pore size of the constructs, the mechanics of the hybrid constructs can be adjusted.^[^ ^33^, ^34^ ^]^ However, a significant limitation of FDM reinforcement is the large fiber diameter, typically ≈200 µm, as these large fibers and pore sizes negatively affect cell adhesion, ‐growth, and ‐differentiation.^[^ ^34^, ^35^ ^]^ Consequently, this approach can lead to stress shielding, where applied forces are primarily absorbed by the reinforcement fibers, thereby potentially failing to stimulate cellular mechanotransduction.^[^ ^36^ ^]^ An alternative to FDM reinforcement is melt electrowriting (MEW). This technique uses a high voltage to accelerate and stretch a thermoplastic polymer, producing fibers ranging in diameter typically from 2 to 50 µm.^[^ ^37^ ^]^ The combination of MEW reinforcement boxes with (3D printing of) hydrogel‐based bioinks has resulted in a new biofabrication process that generates small‐scale (∅6 mm, 7.2 mm total height – 1.2 mm for the cartilage phase) reinforced implants with homogenous mechanical properties approaching the mechanical properties of the native AC, of which the efficacy has already been demonstrated in orthotopic in vivo applications.^[^ ^38^, ^39^, ^40^, ^41^, ^42^, ^43^ ^]^ However, these applications lack the size required for clinical use and the customizability in shape and mechanical design.

The underlying mechanisms of the reinforcement of hydrogel‐based scaffolds with box shaped reinforcement can be attributed to the fiber stretching due to the lateral expansion of the hydrogel under compression, which plays a significant role. This so‐called Poisson effect causes the fiber to be pulled in tension, enhancing the overall stiffness of the composite.^[^ ^44^ ^]^ In addition, the load transfer through the scaffold interconnections and the prevention of buckling increases the overall compressive stiffness.^[^ ^44^ ^]^ The fiber interconnections are structurally more resistant than individual fiber stacks and effectively distribute the compressive loads, preventing the scaffold from collapsing and maintaining the structural integrity of the composite. Finally, the MEW mesh enhances load‐induced fluid pressurization within the hydrogel by reducing the hydraulic permeability. Fluids are retained under pressure by the MEW box scaffold, leading to increased internal fluid pressure during mechanical loading.^[^ ^45^ ^]^ This fluid pressurization is essential for the dynamic mechanical properties of the composite, mimicking the in vivo situation where collagen restricts water swelling caused by proteoglycans.

In the knee joint, distinct zones across the surface of the AC, each exhibiting different compressive properties that highlight the anisotropic nature of the surface.^[^ ^46^, ^47^, ^48^, ^49^, ^50^, ^51^ ^]^ This is also represented in the collagen alignment in the surface, showing different behavior and alignment between loaded and unloaded areas.^[^ ^17^ ^]^ For an optimal integration of the fabricated cartilage constructs, the printed constructs should mechanically match the native cartilage to prevent stress shielding in the surrounding, opposite, and the fabricated tissue. Therefore, this study focuses on matching the local mechanical properties of fabricated cartilage constructs, aiming to address larger‐sized defects.

## 2 Results and Discussion

### 2.1 Production of Anisotropic MEW Scaffolds

**Figure 1:**
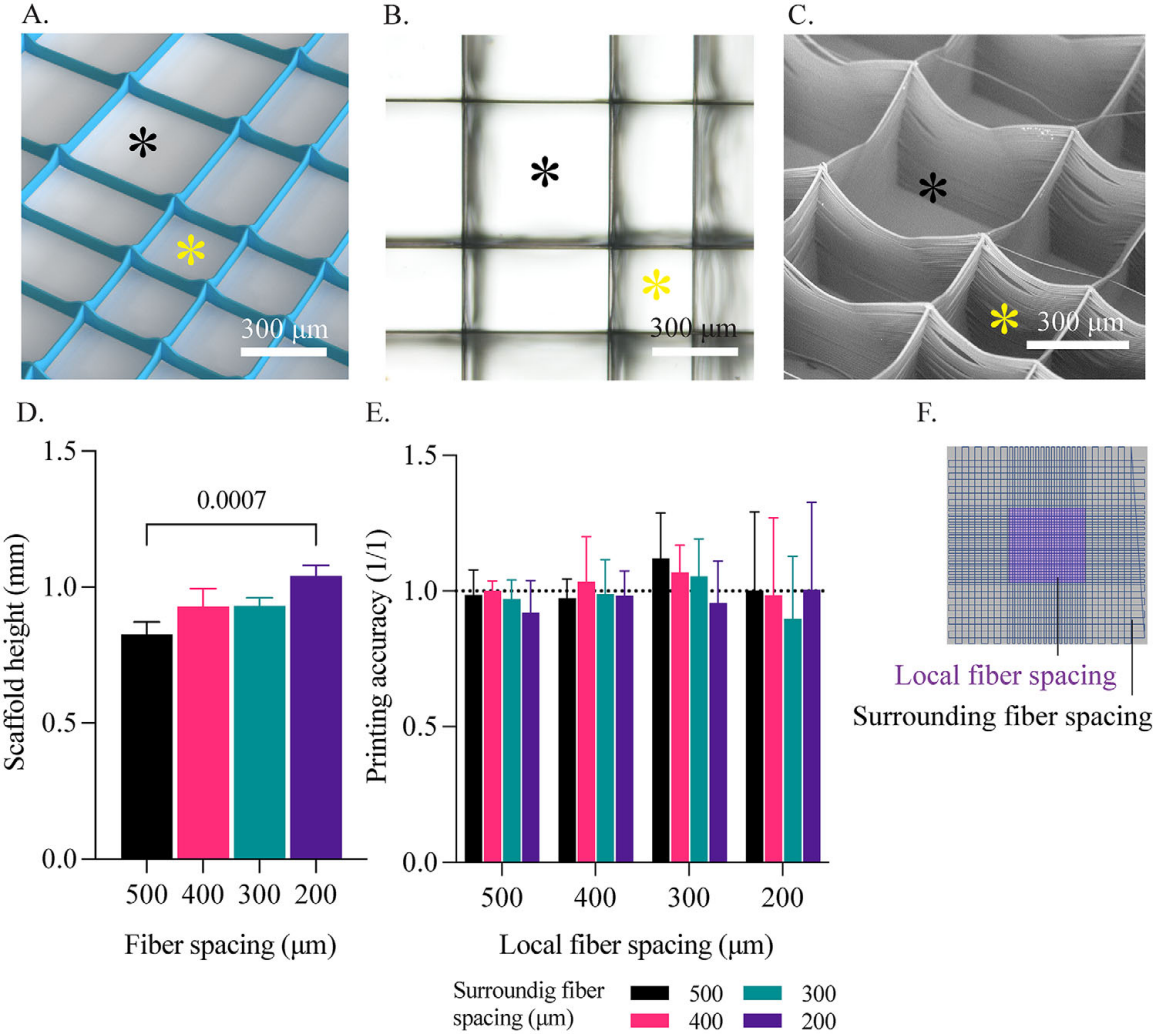
Printability of anisotropic scaffolds with fiber spacing ranging from 500 µm down to 200 µm. A) 3D render of the anisotropic scaffold design, featuring 300 µm inner and 500 µm outer fiber spacing with a height of 10 layers. B,C) Brightfield microscopy and scanning electr imaging of an anisotropic scaffold with 500 µm inner and 300 µm outer fiber spacing. D) Measured construct height for different fiber spacing scaffolds. E) Printing accuracy of anisotropic scaffolds and different fiber spacing scaffolds.

Anisotropic scaffolds of 100 layers in thickness were printed using MEW and reflected the expected model designs (**Figure** **1**A–C). Dedicated inner‐ and outer zones could be distinguished through the different fiber spacings. The overall design of the scaffolds also resulted in transitional zones where the pores shifted from a square to a rectangular shape. These transitional zones with different box structures likely impact the mechanical properties and behavior of the final constructs. A statistically significant difference between the height of the 500 (820 ± 47 µm) and 200 µm (1040 ± 40 µm) fiber spacing scaffold structures was observed (Figure 1D). This can be explained by charge building up in the scaffold, especially when the total scaffold height increases and the structure gets more complex, as is the case for the 200 µm spacing scaffolds. This can result in more bridging and defects in the top layers of the scaffold, increasing the total height.^[^ ^52^ ^]^ The surface area of the fabricated boxes did reflect the designed surface area, representing a printing accuracy of 0.97 ± 0.19. The root mean square error (RMSE) increased for fiber spacing reading 200 µm, but no significant difference was found between the groups (Figure S4, Supporting Information). Therefore, the anisotropic scaffolds could be printed with the same accuracy and repeatability as homogenous scaffold designs, in line with other reports on MEW scaffolds.^[^ ^53^, ^54^ ^]^

### 2.2 Mechanical Properties

**Figure 2:**
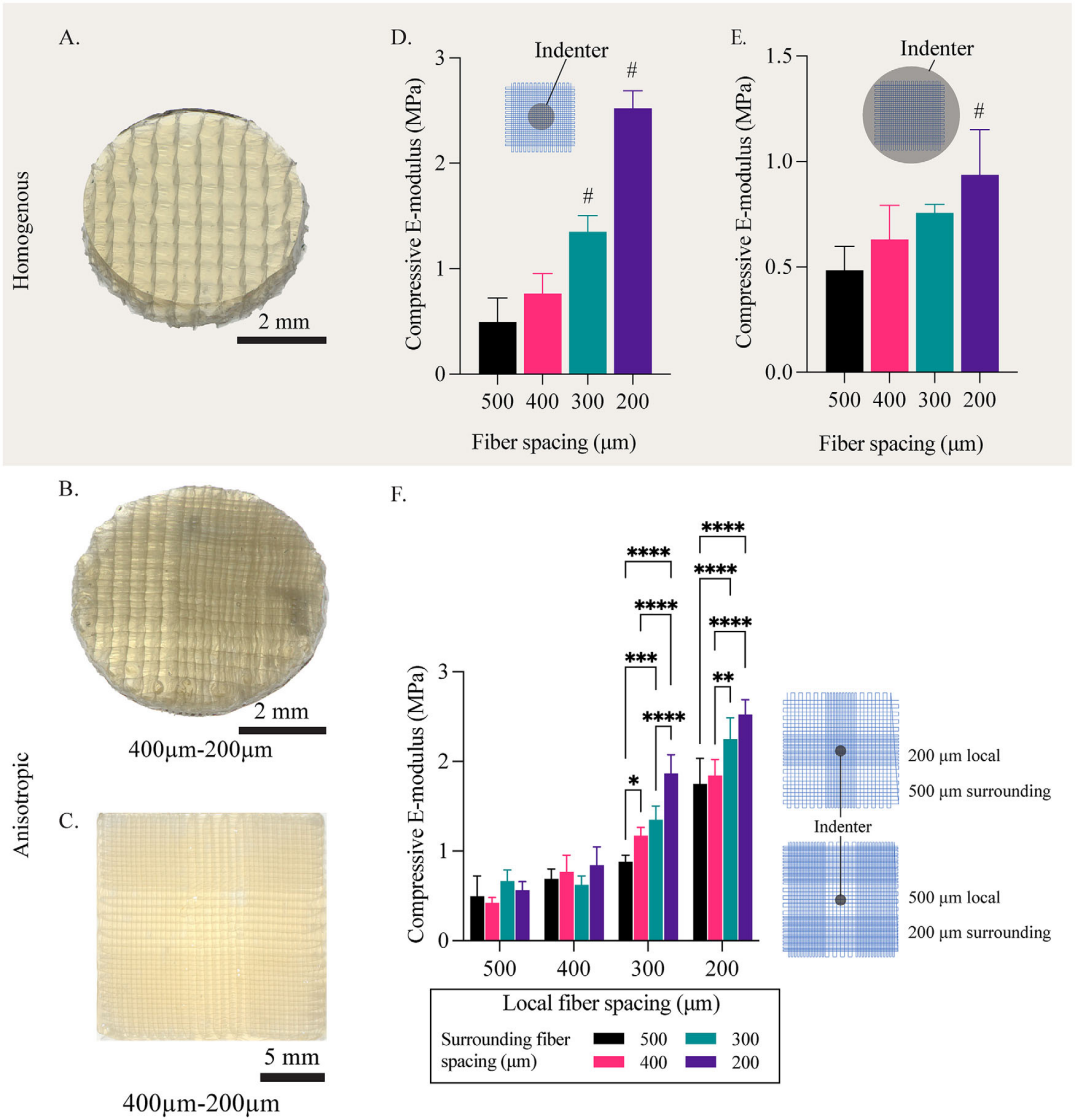
Mechanical properties of anisotropic scaffold and the effect of fiber spacing on the E‐modulus of the constructs after casting in gelMA. A,B) Stereomicroscopy image of a disk construct from a homogenous and anisotropic scaffold. C) Full‐size anisotropic scaffold with 200 µm inner fiber spacing and 400 µm surrounding fiber spacing casted in gelMA. D) E‐modulus for different fiber spacings when a construct is compressed locally. # significant with all other groups. E) E‐modulus for different fiber spacings for bulk compression. # significant with all other groups *p* ≤ 0.05. F) E‐modulus of anisotropic scaffold when compressed with local indentation. ^*^ *p* ≤ 0.05, ^**^ *p* ≤ 0.01, ^***^ *p* ≤ 0.001, ^****^ *p* ≤ 0.000.

Both homogenous and anisotropic scaffolds were successfully combined with gelMA hydrogels (**Figure** **2**A–C), and the anisotropic scaffold retained a clear distinction between the *local* and *surrounding* zones showing different fiber spacings. After casting and allowing for overnight swelling, the constructs measured an average height of 1.23±0.03 mm. The increase in height is mainly due to the swelling behavior of gelatine methacrylol (gelMA),^[^ ^55^ ^]^ although no swelling in the diameter was detected. This is likely due to the restrictions from the MEW reinforcement, which prevents the construct from swelling in this direction, resulting in a thin layer of non‐reinforced gelMA on top of the construct. The construct's thickness is ≈60% of native human AC and 80% compared to equine tissue.^[^ ^3^ ^]^ The constructs were analyzed for mechanical properties with a dynamic mechanical analyzer (DMA), which showed linear behavior after the first −3% strain up to −30% of total strain. This linear behavior suggests that the pre‐load and initial strain do take out the variability of the non‐reinforced gelMA on top of the construct. The mechanical compression data showed that the fiber spacing did affect the compressive mechanical properties, showing a negative linear relation in which the compressive E‐modulus increases with decreasing fiber spacing. This is applied to both local‐ and bulk compression (Figure 2D,E). For local compression, the compressive modulus ranged from 0.49 ± 0.18 MPa to 2.52 ± 0.17 MPa (509% increase) for fiber spacings of 500 and 200 µm, respectively. In contrast, for bulk compression, the difference was less pronounced, with values ranging from 0.50 ± 0.2 MPa to 0.94 ± 0.16 MPa (193% increase) for 500 and 200 µm, respectively. Native cartilage showed an average compressive modulus for bulk compression of 2 MPa.^[^ ^56^ ^]^ The difference between local and bulk compression suggested that, in the case of local compression, the surrounding area was indirectly affected during compression. This could be attributed to the buckling behavior and trapped water components within the MEW meshes, increasing the hydrostatic pressure.^[^ ^53^ ^]^ During bulk compression, the sample could expand laterally as it was not confined. However, in local compression, the compressed area was constrained by the surrounding construct, leading to higher hydrostatic pressures from the sides and resulting in a different mechanical behavior under compression. This variation could also influence the mechanical properties of the large‐sized construct, where the borders exhibited lower overall mechanical stiffness due to the lack of hydrostatic pressures. The observed influence of variations in fiber spacing on the mechanical properties of the area is in line with those in previous investigations.^[^ ^53^ ^]^

The effect of the *surrounding* region was most predominant in samples with *local* fiber spacing of 300 and 200 µm (Figure 2F). For those groups, the *local* mechanical properties showed significant differences in response to changes in the *surrounding* fiber spacing. Constructs with the same *local* fiber spacing (200 µm), showed an E‐modulus ranging from 1.7 up to 2.5 MPa for a *surrounding* fiber spacing of 500 and 200 µm, respectively. This increase represents 70%, which was only caused by altering the fiber spacing, outside the compressed area (Figure 2F). For reinforced constructs with fiber spacing of 500 and 400 µm no significant differences between homogenous and anisotropic scaffold reinforcements were observed for bulk and local compression.

### 2.3 Internal Calculated Stresses

**Figure 3:**
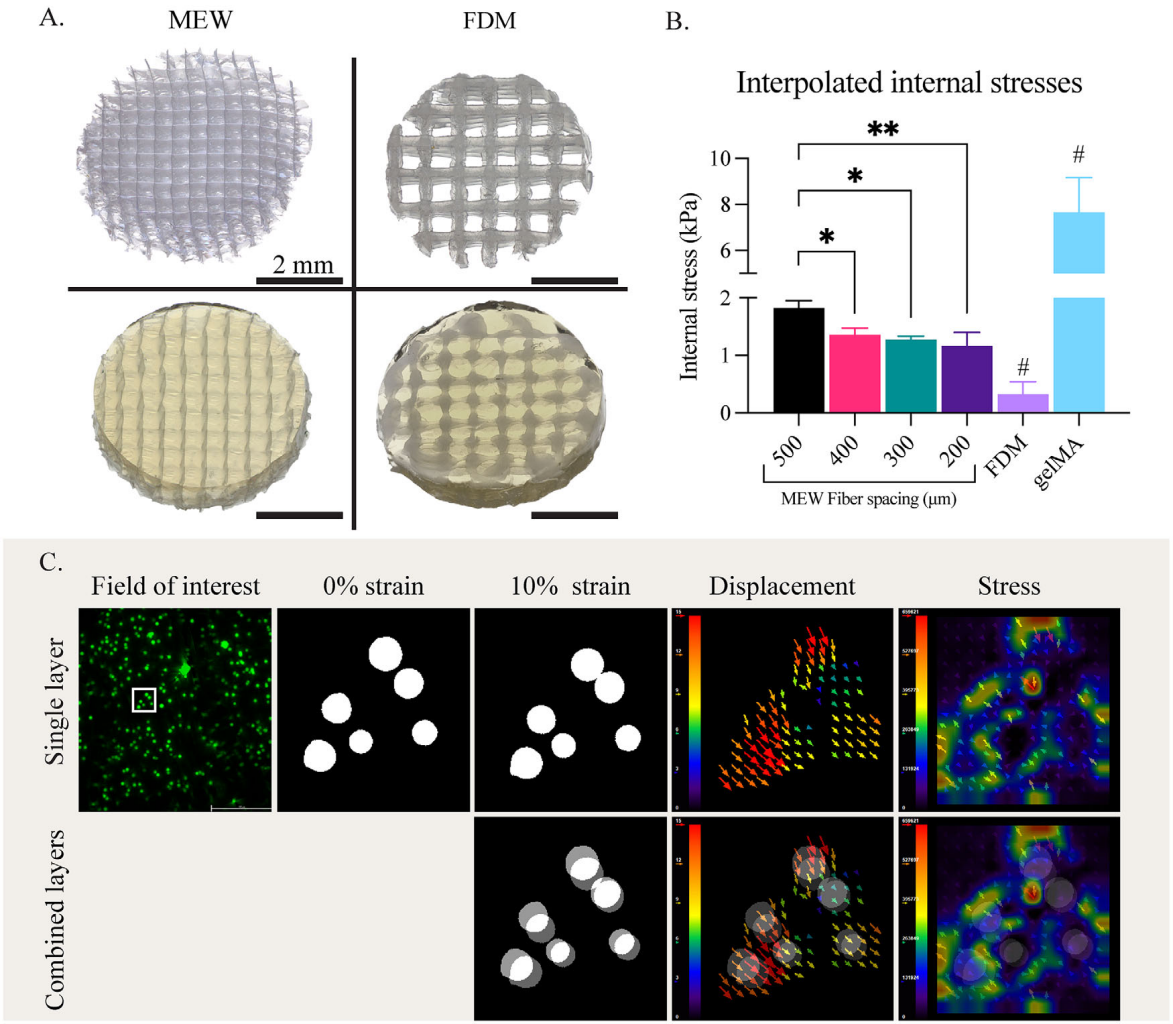
Traction force microscopy (TFM) data from different fiber spacing samples. A) Stereomicroscopy images of MEW and FDM samples before and after casting of gelMA. B) Internal stresses interpolated by TFM. # Significant to all other groups *p* ≤ 0.05, ^*^ *p* ≤ 0.05, ^**^ *p* ≤ 0.01. C) Analysis steps when applying TFM. Showing a focused area for visualization, comparing no strain and 10% strain conditions, visualizing displacement and interpolated stresses by the FTTC plugin.

Constructs with MEW, FDM, and without reinforcement were successfully cast with gelMA containing fluorescent microbeads (**Figure** **3**A). The constructs measured a height of 1 mm. As the FDM reinforcement took more polycaprolactone (PCL) volume due to the thicker strands, the remaining volume of gelMA was smaller compared to MEW and gelMA only samples. However, the measured area – in between the strands – contained the same amount of fluorescent microbeads as the ratio within the gelMA was kept constant. Tracking of the beads, with and without compression, resulted in a displacement map where the two different states were compared. As expected, the gelMA only constructs interpolated the highest internal stresses of 7.64 ± 1.18 kPa. FDM reinforced constructs showed the lowest internal stresses with a value of 0.32 ± 0.16 kPa. MEW reinforced samples exhibited a significantly higher stress for the 500 µm group, but not between any of the other fiber spacings (Figure 3B). The internal stresses within MEW‐reinforced constructs varied from 1.8 ± 0.1 kPa (at 500 µm fiber spacing) to 1.2 ± 0.2 kPa (at 200 µm fiber spacing). In all the samples, the displacement of the beads could be successfully tracked (Figure 3C).

Upon examining the internal stresses, we observed that FDM reinforcement exhibited stress shielding, with less than 4 ± 2% of the original stresses remaining within the gel. In contrast, microfiber‐reinforced constructs retained 19 ± 3% of the original stresses. Notably, the 500 µm fiber spacing group exhibited significantly higher stress levels compared to other fiber spacing groups. This data confirmed that the reinforcement effectively took up part of the load from the hydrogel, as anticipated. Specifically, the FDM reinforcement absorbed nearly the entire load, whereas the MEW reinforcement bore most of the load while still permitting internal stresses within the gel.

Physiological loading positively influences cartilage matrix production by chondrocytes. The cellular response to mechanical stimuli varies according to the type of force applied (compression, shear, or multidirectional) and the duration of the stimulus. Adequate mechanical stimulation is crucial for constructing cartilage implants that support proper chondrocyte function. Studies have shown that chondrocytes require mechanical stimulation for effective matrix synthesis.^[^ ^57^, ^58^, ^59^ ^]^ Applying a pressure of 1 kPa for 30 mins up‐regulates matrix remodeling enzymes, followed by an increase in collagen type II and aggrecan gene expression compared to unstimulated samples.^[^ ^60^ ^]^ This 1 kPa stimulation is consistent with the internal stresses interpolated by traction force microscopy (TFM) in the MEW reinforced constructs for the 400, 300, and 200 µm groups.

The compressive E‐modulus of MEW‐reinforced gelMA constructs is lower than that of native cartilage and FDM‐reinforced gelMA, where native AC shows a E‐modulus of ≈2 MPa.^[^ ^56^ ^]^ However, this assessment does not consider the presence of cells and their ECM production, which would add further stability.^[^ ^61^ ^]^ Given the measured internal stresses, these constructs should provide sufficient mechanical stimulation for the cells to produce an ECM with potential load‐bearing functionality. Whilst the FDM‐reinforced gelMA exhibited higher compressive properties than native tissue, it does not permit mechanical stimulation of the hydrogel‐embedded cells. Additionally, the higher compressive modulus could also lead to stress shielding of the surrounding tissue post‐implantation.

### 2.4 Generation of the Constructs of Clinically Relevant Sizes

#### 2.4.1 Mechanical Properties

**Figure 4:**
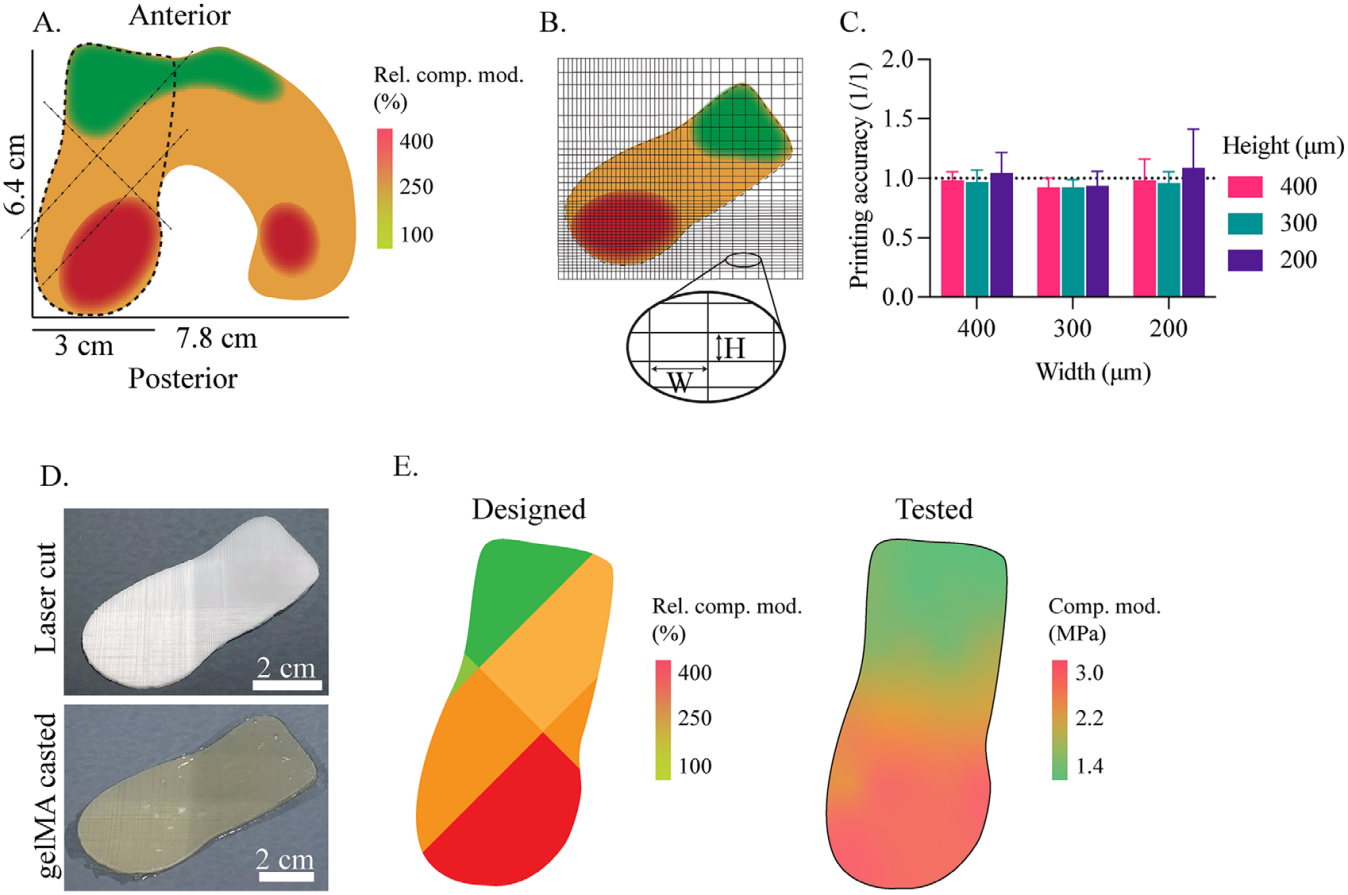
Large‐size cartilage construct based on the mechanical properties of the articular cartilage on the lateral femoral condyle of the right knee. A) Heatmap generated with the relative compressive properties based on data from the literature.^[^ ^46^, ^47^, ^48^, ^49^ ^]^ B) MEW scaffold design to reflect the different mechanical zones of the femoral condyle. C) Printing accuracy of the different mechanical zones. D) Scaffold after laser cutting and casting with gelMA. E) Relative compressive properties of the different zones of the condyle MEW mesh.

The design of the femoral condyle compression heatmap was developed using values from literature (**Figure** **4**A). The variation between the different areas was shown in relative values. A large scaffold was fabricated, achieving an accuracy of 0.98 ± 0.15 across the different fiber spacing zones (Figure 4B,C), consistent with previous anisotropic scaffolds (Figure 1). The scaffold was then laser cut into the desired shape and combined with gelMA (Figure 4D), resulting in a flat condyle‐shaped construct with distinct fiber‐spacing zones and an overall thickness of 1 mm. These varying fiber densities correspond to different mechanical properties. Areas with smaller fiber spacing, and thus higher fiber density, exhibited greater mechanical stiffness compared to areas with lower fiber density, which was shown earlier in this study and still applied to the free‐form construct (Figure 4E). Overall, the anisotropic scaffold showed a variation in E‐modulus ranging from 1.4 up to 3.0 MPa. Thus, more than twice the compressive E‐modulus was achieved within a single construct, while reflecting the variations as designed. Native AC is anchored to the subchondral bone, preventing any translation of the chondral layer. In this research, the large anisotropic construct was placed inside a frame (Figure S3, Supporting Information), which restricted lateral expansion, thereby mimicking the native anchoring. Since gelMA is water‐based, it can be considered incompressible. In the native environment, AC exchanges fluids with the synovial fluid.^[^ ^15^ ^]^ The same effect occurs with the reinforced gelMA constructs. However, due to the size of the indenter relative to the total volume of the construct, this effect is negligibly small.

Although anisotropic mechanical properties of scaffolds have been documented in the literature, particularly in cardiac and vascular applications, these properties are typically achieved by tailoring a homogeneous design.^[^ ^62^, ^63^ ^]^ Consequently, under tension, the mechanical properties do not exhibit linear behavior. This approach results in constructs with uniform mechanical properties throughout, lacking the ability for anisotropic mechanical design. For meniscus regeneration, an anisotropic approach has demonstrated the creation of distinct mechanical zones by combining different fiber spacings within a single construct.^[^ ^64^ ^]^ However, this method is limited to just two distinct zones. Additionally, this approach requires that the fiber spacings be aligned such that one is a multiple of the other (e.g., 400 µm – 200 µm and 600 µm – 300 µm – 200 µm), allowing the fibers to blend seamlessly. Therefore, the customization is restricted to specific combinations of fiber spacings.

#### 2.4.2 Cellular Behavior and ECM Deposition in Anisotropic Scaffolds

**Figure 5:**
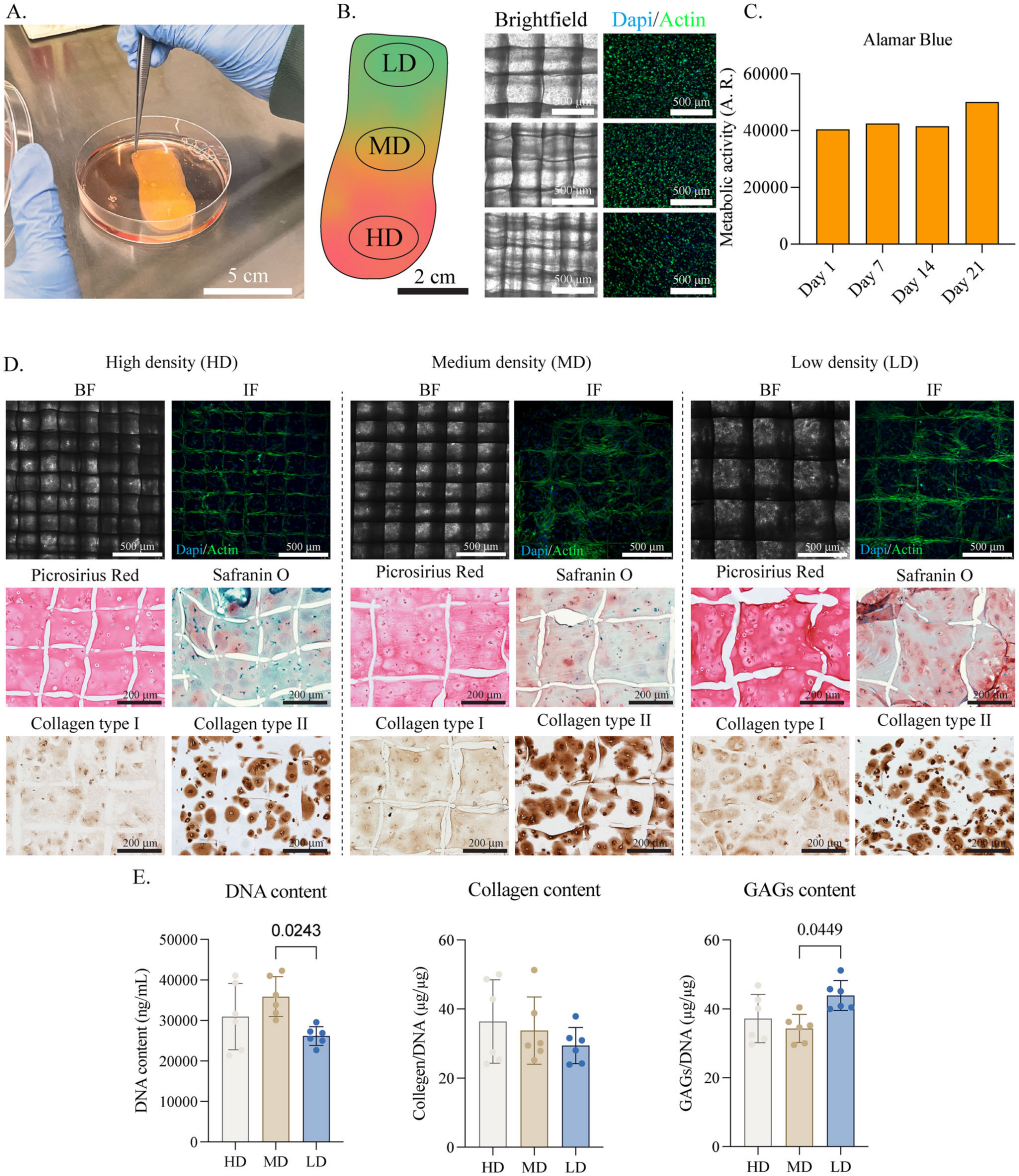
In vitro validation of large‐scale, anisotropic scaffolds. A) Culture of large‐sized, anisotropic scaffold design with cell‐laden gelMA after casting. B) Brightfield images and nuclei (DAPI)/actin immunofluorescence staining of different zones directly after seeding. C) Alamar blue measurements from the entire construct. D) Brightfield imaging and nuclei (DAPI)/actin immunofluorescence staining after 28 days of culture; Picrosirius red, safranin‐O, and collagen type I and type II immunostaining after 28 days of culture. E) DNA (ng/ml), collagen per DNA (µg/µg), and GAGs per DNA (µg/µg) content between low (LD), medium (MD), and high‐density (HD) zones.

Large anisotropic scaffolds were successfully combined with articulair cartilage progenitor cells (ACPC)‐laden gelMA and cultured for up to 28 days in chondrogenic differentiation media (**Figure** **5**A). The fiber spacings within the construct remained unchanged and distinguishable after hydrogel casting, with a uniform cell distribution observed after cell seeding (Figure 5B). The metabolic activity of the cells remained stable throughout the initial culture period, indicating that the cells adapted to the 3D environment and maintained metabolic function upon initial cell casting, likely due to the supportive, biomimetic properties of the hydrogel. The plateau in metabolic activity may reflect reduced proliferation as a result of cell differentiation (Figure 5C). After 28 days of chondrogenesis, ACPCs were evenly distributed throughout the construct, predominantly attached to and aggregated around the MEW microfibers (Figure 5D). Notably, at large fiber spacing (400 µm), ACPCs tended to cluster along the box corners. In contrast, at smaller fiber spacings (300 and 200 µm), the cells predominantly adhered to the denser PCL network, forming a uniform layer that covered the spaces between the fibers. ACPCs exhibit an elongated morphology but retain the capacity to produce collagen type II and glycosaminoglycans during culture in chondrogenic differentiation media (Figure 5D). Collagen deposition was confirmed via collagen type I and II immunohistochemistry. Due to GelMA containing collagen type I, most of the constructs also showed slight positive staining for picrosirius red. Collagen type II secreted by the chondrogenically differentiated ACPCs was observed surrounding the cells, and similarly, GAGs were largely deposited in the pericellular region. Previous studies have shown that ACPCs cultured in gelMA progressively increase the production of ECM components, such as GAGs.^[^ ^24^, ^65^ ^]^ Therefore, it is expected that extending the culture period would lead to a greater accumulation of GAGs and collagen type II throughout the construct. Although ECM production over time will contribute to improved mechanical properties, its effect at this stage of differentiation (28‐days) remains limited compared to the mechanical reinforcement provided by the MEW fiber network.^[^ ^24^ ^]^ Additionally, no significant differences in total collagen content were observed between the different zones of the scaffold (Figure 5E).

The overall DNA content was significantly lower in the areas with low fiber density compared to those with medium density, indicating a lower cell content at day 28 (Figure 5E), despite the construct being casted with a homogeneous cell suspension. Still, DNA content allowed normalizing of other components of the ECM. The GAGs/DNA ratio showed higher GAG content in the lower density zone.^[^ ^63^ ^]^

## 3 Discussion

The large‐sized bio‐constructs fabricated in this study exhibited anisotropic mechanical properties across different zones, mirroring the anisotropic mechanical behavior observed in native cartilage. In this study, cell‐laden gelMA was added in a manual process. By combining MEW with extrusion‐based bioprinting (EBBP), an automated process for biofabrication of large, mechanically customized cartilage constructs would be possible. It has been shown that the convergence of these two techniques allows for greater complexity in the spatial deposition of cells within the constructs.^[^ ^42^, ^43^, ^66^ ^]^ Additionally, the anisotropic design can be integrated with a multi‐axis (5‐ or 6‐axis) printing setup which increases the reachability for complex 3D substrates.^[^ ^67^, ^68^ ^]^ Especially the 6‐axis robotic printer shows the greatest potential, as it offers freedom in both the linear axis (x, y, and z) and the rotational axis (roll, pitch, and yaw), increasing reachability for convex and concave surfaces, such as the femoral condyles, trochlea, and patella. The increased reachability would allow to print directly onto a 3D structure representing a femoral condyle, created from a osteoinductive ceramic to function as a bone anchor, needed for stable implants, while replicating the distinct mechanical zones.^[^ ^56^, ^69^, ^70^ ^]^ This would result in a patient specific implant, without the need for any adjustments during surgery, as can be case for other approaches utilizing a universal design.^[^ ^71^ ^]^

From a biological perspective, this study demonstrated homogeneous cell behavior within the anisotropic scaffold. ACPCs mainly attached and aggregated around the MEW microfibers at larger fiber spacings, while at smaller fiber spacings, they formed a more uniform layer by bridging the fibers. This behavior is consistent with similar findings reported in previous literature.^[^ ^72^, ^73^ ^]^ ECM production was homogeneous in the distinct zones of the construct. Yet, the area with lower fiber density had a significantly higher concentration of GAGs. This may result from the fact that larger pores provide more space for ECM deposition, especially important for GAGs due to their water‐attracting and swelling properties. As GAGs accumulate, the larger pores may leave more room for swelling to occur without being constrained by the fibers. In addition, cell aggregation is an important signal for the phenotypic expression of chondrocyte markers, which was also observed to occur more frequently in large fiber spacing.^[^ ^74^ ^]^ However, several aspects still need to be further explored. First, mechanical loading was not included in this study. To further differentiate and mature the tissue during culture, mechanical stimulation (e.g., by a bioreactor representative of the in vivo situation) could enhance matrix production.^[^ ^75^ ^]^ Mechanical stimulation is important as chondrocytes, the primary cells in cartilage, detect mechanical stimuli through mechanotransduction sensors and require this to maintain their normal function, ECM synthesis, and overall cartilage homeostasis.^[^ ^76^ ^]^ In AC implants, load distribution significantly affects the behavior of chondrocytes seeded onto these constructs.^[^ ^77^ ^]^ Further exploration of the mechanical stimulation of cell‐seeded constructs and ECM production at different pore sizes would be insightful. With TFM, it was confirmed for the first time that FDM reinforcement indeed results in stress shielding, where the MEW reinforcement allowed for internal stresses. If the scaffold absorbs all the mechanical loading, chondrocytes are shielded from the mechanical stresses they would typically experience in their native environment.^[^ ^78^, ^79^ ^]^ This deprivation of appropriate mechanical signals leads to changes in their metabolic activity and matrix production, resulting in the production of mechanically inferior fibrocartilage instead of hyaline cartilage.^[^ ^80^ ^]^ MEW reinforcement, on the other hand, allows for adequate internal stress loading within the gel of ≈1 KPa, which has been shown to promote collagen type II and aggrecan gene expression. In contrast, FDM reinforcement only provides ≈300 Pa of internal stress loading.^[^ ^60^ ^]^

For long‐term stabilization, the biodegradable composition of the materials used (PCL and gelMA) should be considered. Initially, the reinforced gelMA provides most of the support when it has not yet been replaced by the ECM produced by the differentiated cells. Histology samples indicate that the produced ECM is unorganized and not comparable to the highly organized native tissue.^[^ ^56^ ^]^ Therefore, as gelMA degrades over time, there is a risk of reduced mechanical stability. The MEW scaffold offers longer‐term stability but also exhibits significant in vivo degradation after the first year of implantation.^[^ ^81^ ^]^ To improve long‐term stability, future research should explore strategies to enhance collagen alignment both across the surface and throughout the depth of the tissue to support the formation of native‐like AC tissue for long‐term stability.

## 4 Conclusion

This study demonstrates for the first time the successful fabrication and maturation of large sized, mechanically anisotropic cartilage tissue constructs with tailorable local mechanical properties and living cartilage matrix producing cells. As the fibers interact with the environment, local adjustments also affect the surrounding area's mechanical stiffness. This effect can be leveraged to create a custom mechanical loading design of the reinforced hydrogel construct. Homogenous cell behavior is demonstrated within the different zones of the construct and the cells do not exhibit stress shielding. The cells are viable and produce GAGs and ECM, including collagen type II.

Previous orthotopic in vivo studies already confirmed the mechanical stability of small‐diameter reinforced constructs. This study now demonstrates the feasibility of upscaling such implants to large sizes, offering the possibility to cover an entire femoral condyle while maintaining customizability in mechanical behavior and homogenous cell response.

## 5 Experimental Section

### Methods

This study investigated the mechanical properties of MEW‐reinforced GelMA constructs, emphasizing the effects of varying fiber spacing and anisotropic designs. Homogeneous and anisotropic scaffolds were fabricated and embedded in GelMA, followed by detailed imaging and mechanical analysis. Large cartilage constructs with distinct mechanical regions were created, measured, and seeded with chondroprogenitor cells. The constructs were cultured to assess cellular behavior and mechanical performance. Comprehensive scaffold and cell characterization were conducted to evaluate the efficacy of the constructs.

### Fabrication of MEW Scaffolds—Printer Settings

MEW scaffolds were generated by printing PCL (Corbion, The Netherlands) on a 1 mm thick glass slide using a Discovery1 bioprinter system (RegenHU, Switzerland). PCL was heated to 80 °C and extruded through a 24 G nozzle. A voltage range of 5–5.5 kV, a pressure of 0.1 MPa, a collector distance of 4 mm, and a speed range of 8–12 mm s^−1^ were applied. Custom G‐code was used to control the tool head. After printing, the scaffolds were sprayed with 70% ethanol and gently released from the glass slide. Any excess ethanol was allowed to evaporate.

### Fabrication of MEW Scaffolds—Scaffold Design

Homogeneous scaffolds were fabricated using a box design, consisting of 100 layers with a fiber diameter of 10 µm. The inter‐box spacing varied from 500 to 200 µm in 100 µm increments. This range was selected based on prior research indicating the optimal porosity of MEW meshes for GelMA reinforcement, as well as previous in vivo studies demonstrating the ideal fiber spacing for reinforcement within this range.^[^ ^53^, ^56^ ^]^ Fiber spacing exceeding 500 µm lacks any mechanical support, and below 200 µm the printability is limited with the desired fiber diameter. The scaffolds used for mechanical analysis measured 15 × 15 mm in overall dimensions with a thickness of 1 mm. Additionally, anisotropic scaffolds were produced, featuring square and rectangular box patterns, also comprising 100 layers with a fiber diameter of 10 µm. The scaffolds were divided into two regions: the inner or center section measuring 5 × 5 mm (referred to as “*local*”) and an outer section composed of surrounding fibers, with a total size of 15 × 15 mm (referred to as “*surrounding*”) (Figure S1, Supporting Information). Combinations of *local* and *surrounding* fibers were used from 500, 400, 300, and 200 µm, combining each for both zones, resulting in 16 groups.

### Fabrication of MEW Scaffolds—Imaging

Samples were imaged using brightfield microscopy at 4‐ and 10‐times magnification (Olympus BX43) to assess printability and scanning electron microscopy (SEM, Phenoms Pro, ThermoFischer) for a detailed overview of the construct, operating at acceleration voltages of 5 kV and 10 kV.

### Fabrication of FDM Scaffolds—Printer settings

Fused deposition modeling PCL (Corbion, The Netherlands) scaffolds were fabricated using a Discovery1 bioprinter system (RegenHU, Switzerland). The PCL container was heated to 90 °C and extruded at 80 °C through a 0.2 mm diameter nozzle with an extrusion rate of 3 rotations per minute and a print speed of 5 mm s^−1^.

### Fabrication of FDM Scaffolds—Scaffold Design

The FDM scaffolds were disk‐shaped with a diameter of 6 mm, featuring a 500 µm rectilinear infill pattern and 200 µm diameter fibers, resulting in a fiber spacing of 300 µm. The scaffolds consisted of 5 layers of 200 µm, creating a total height of 1 mm.

### Gelatin Methacryloyl

GelMA with a degree of functionalization of 80% was synthesized based on previously described protocols.^[^ ^82^ ^]^ To generate the gelMA hydrogels, 0.95 mL of a 0.1g mL^−1^ gelMA solution was prepared in PBS. Additionally, 0.025 mL of 2.5% 5 mм sodium persulfate (SPS, Sigma–Aldrich, USA) solution was added as a crosslinker (2.5% v v^−1^). This was followed by the addition of 2.5% 0.5 mм dichloro‐ruthenium (II) hexahydrate (RU, Sigma–Aldrich, USA).

### Embedding of the MEW Scaffolds in gelMA

Homogenous scaffold designs (equal fiber spacing throughout) were sampled using a 6 mm biopsy punch (Razormed, BAP medical) from the large scaffolds. The scaffolds were then placed in custom‐made Teflon casting molds.^[^ ^42^, ^83^ ^]^ For the anisotropic scaffolds, custom‐designed molds were 3D‐printed on a X1 Carbon FDM printer (Bambu lab, China) for gelMA casting. GelMA (10% w v^−1^) was added to the scaffolds and crosslinked using a 20‐Watt white light LED (Jobmate, Australia) for 7 min at a height of 10 cm. The samples were stored at 4 °C overnight in PBS to allow swelling of the gelMA and imaged using stereo microscopy (Olympus SZ61). Additionally, FDM reinforced constructs and gelMA only constructs were cast as controls for traction force microscopy, following the same procedure. After mechanical analysis, samples were imaged using a stereomicroscope (Olympus SZ61).

### Fabrication of Large Anisotropic Constructs

The design of the large constructs that reflect the mechanical anisotropy, as observed within native AC of the femoral condyle, was based on mechanical data obtained from the literature.^[^ ^46^, ^47^, ^48^, ^49^ ^]^ The scaffold was printed as part of a large square‐shaped construct and subsequently cut to shape using a Fusion Pro laser cutter (Epiloglaser, the Netherlands) set to 5% power, 100% speed, and a frequency of 50%. The scaffold contained three zones: high density (HD 200 µm fiber spacing) medium density (MD, 300 µm fiber spacing), and low density (LD, 400 µm fiber spacing). The anterior side of the joint, characterized by low mechanical properties, corresponded to a low fiber density. The lateral side, exhibiting high stiffness, was represented by a high fiber density, with a transition zone (medium density) in between. The MEW scaffold was positioned in a custom Teflon mold, which was enclosed with glass slides on both the upper and lower surfaces (Figure S2, Supporting Information), and cast with gelMA.

### Cell Culture—Cell Isolation, Seeding, and Culture Conditions

Articular Cartilage Chondroprogenitor cells were isolated from the healthy knee joints of deceased mature equine donors, as previously described.^[^ ^24^ ^]^ Briefly, ACPCs were first selected based on their adhesion to fibronectin and their colony formation capacity. Cells were then expanded in expansion growth medium DMEM‐high glucose‐ GlutaMAX‐pyruvate (Gibco, 31966) supplemented with 10% fetal bovine serum (FBS; Westburg, CA FBS‐HI‐11A), 1% penicillin/streptomycin (Gibco, 15140‐122), 1% ascorbic acid 2‐phosphate (Merck A8960), 1% non‐essential amino acids (Gibco, 11140050), and 5 µL mL^−1^ basic fibroblast growth factor (bFGF; Bio‐Techne, 233‐FB) until passage 3. After expansion, ACPCs were embedded in 80% DoF gelMA at a concentration of 20 × 10^6^ cells/mL by adding the cells to the hydrogel and mixing with a positive displacement pipette until a homogenous suspension was achieved. The anisotropic MEW scaffolds were cast with 2 mL of the cell‐laden gelMA, crosslinked as previously described and cultured for 28 days in chondrogenic differentiation medium consisting of DMEM‐high glucose‐ GlutaMAX‐pyruvate (Gibco, 31966) supplemented with 1% ITS Premix (Corning, 354352), 1% penicillin/streptomycin (Gibco, 15140‐122), 1% ascorbic acid 2‐phosphate, 1% HEPES buffer (1м; Gibco, 15630080), 0.4% dexamethasone (0.1  ×  10^−6^ м Sigma–Aldrich, D8893) and 0.1% recombinant human transforming growth factor β (TGFβ1) (10 ng/mL, Prepotech,100‐21). Media was refreshed twice a week, as previously described.^[^ ^24^ ^]^ During culture, the samples were imaged using brightfield microscopy (Olympus BX43).

### Scaffold Analysis—Mechanical Analysis

GelMA‐embedded MEW scaffolds were tested using a (DMA (TA instruments Q800). A preload of 0.001 N was applied to make sufficient contact between the indenter and the sample and to measure the height of the sample. The strain ramp was then set to ‐20%/min from 0% to ‐30% strain, while the stress was simultaneously measured every 0.5 s. The compressive E‐modulus was measured from the linear region of the stress‐strain curve, between 10% and 15% strain, as this reflects native loading for AC and is in accordance with established testing procedures.^[^ ^84^, ^85^ ^]^ For local compression, a flat indenter with a diameter of 2 mm was used. For bulk compression, a flat intender covering the entire construct (∅25 mm) was used. For the large anisotropic scaffold, the construct was placed in a 3D‐printed holder to prevent any lateral expansion (Figure S3, Supporting Information). The holder featured dedicated areas for the indenter to measure the construct. The same testing protocol, from 0 to ‐30% strain, was applied using a 2 mm flat indenter.

### Scaffold Analysis—Printing Height and Accuracy

The height of the printed scaffolds was measured using the 2 mm flat indenter on the DMA and applying a pre‐load of 0.001 N to ensure proper contact and accurate measurement at the fiber crossing. Printing accuracy was assessed by quantifying the area of the printed boxes using Fiji (ImageJ 1.54f).^[^ ^86^ ^]^ The measured surface area was then compared to the theoretical surface area, and the printing accuracy was calculated using Equation 1. For the anisotropic scaffolds, the local area was used to determine the printing accuracy.

(1) $$Printing\;accuracy=\frac{Measured\;area\;\left(mm^{2}\right)}{Theoretical\;area\;\left(mm^{2}\right)}$$

### Scaffold Analysis—Internal Stressed, Traction Force Microscopy

Traction force microscopy was used to interpolate the internal stresses in the reinforced gelMA constructs.^[^ ^87^, ^88^ ^]^ Fluorescence polystyrene microbeads (∅5 µm, 508 nm excitation wavelength, 35‐2 ThermoFischer) were dispersed in gelMA at a 3 ± 0.2 mg ml^−1^ concentration and vortexed prior to crosslinking to ensure proper distribution of the beads. The compressive forces required to achieve a 10% compression of the 300 µm MEW‐reinforced gelMA samples were calculated using Equation 2. This internal pressure was then uniformly applied to all compressive groups. Utilizing the calculated forces and corresponding stress values from the DMA data, the necessary strain to attain the same compressive stress was determined. A customized compression tool was used to image the samples with and without applied stress using fluorescence microscopy (Thunder, Leica). These images were imported into Fiji (ImageJ 1.54f) and processed as previously described.^[^ ^88^ ^]^ Particle Image Velocimetry (PIV) analyzed the displacement of the beads in the sample by comparing both the compressed and uncompressed states. The Fourier Transform Traction Cytometry (FTTC) method was used to interpolate the internal stresses.^[^ ^88^ ^]^ The PIV and FTTC methods were analyzed with the PIV and FTTC plugin for Fiji.^[^ ^89^ ^]^

(2) $$\sigma =\frac{E}{\varepsilon }$$

### Cell and ECM Characterization—Metabolic Activity

To assess the cell metabolic activity in the samples, an Alamar Blue (Resazurin sodium salt, Alfa Aesar) assay was conducted according to the manufacturer's protocol. The samples were incubated with the Alamar Blue reagent for 4 h at 37 °C. After incubation, fluorescence intensity was measured using a microplate reader (Clariostar, Germany) at an excitation wavelength of 560 nm and an emission wavelength of 590 nm. The metabolic activity was then extrapolated from the fluorescence readings, providing an indication of cell metabolic activity.

### Cell and ECM Characterization—Histology

Samples were taken from the high‐, medium‐, and low‐density zones of the large anisotropic construct by using a 6‐mm biopsy punch. Subsequently, they were fixed in 4% paraformaldehyde for 30 min and dehydrated before paraffin embedding. Sections (5 µm thick) were cut using a microtome (Leica, Germany). Samples were deparaffinized and stained with 0.1% picrosirius red solution (1h) for collagen staining, 1/1 solution Weigert's hematoxylin (10 min) for nuclei staining, and 0.125% safranin‐O (5 min) for GAG staining, the latter counterstained with 0.4% aqueous fast green for 4 min.

For collagen type I and type II immunohistochemistry, Pronase (1 mg mL^−1^) (Merck PRON‐RO) and hyaluronidase (10 mg mL^−1^) (Merck H3884) were used as antigen retrieval after deparaffinization. Sections were blocked with 5% bovine serum albumin and incubated overnight at 4 °C with a collagen type I rabbit antibody (Abcam, ab138492) diluted 1:400 or a collagen type II mouse antibody (DSHB, II‐II6B3) diluted 1:100. Mouse IgG and Rabbit IgG served as a negative control for collagen type I and collagen type II, respectively. For collagen type I, a goat anti‐rabbit horseradish peroxidase‐conjugated secondary antibody (DAKO EnVision+ (K4010)) was incubated for 30 min at room temperature. For collagen type II, a goat anti‐mouse horseradish peroxidase‐conjugated secondary antibody (DAKO PO447) diluted 1:100 was incubated for 1 h at room temperature. Detection was achieved using DAB peroxidase substrate solution (Vector Laboratories, SK‐4100) for 2 min. Nuclei were briefly counterstained with Mayer's hematoxylin. Samples were mounted with xylene‐based, permanent mounting medium (DePeX, 06522) and imaged using brightfield microscopy (Olympus BX51).

### Cell and ECM Characterization—Immunofluorescent Staining of Actin Filaments

Samples were fixed in 4% paraformaldehyde for 30 min and permeabilized with 0.3% Triton X‐100. Following permeabilization, samples were blocked with 5% bovine serum albumin for 30 min and incubated with Alexa Fluor 488 phalloidin (ThermoFisher) for 30 min to stain actin filaments. Nuclei were counterstained with DAPI (Sigma‐Aldrich) at a 1:1000 dilution. The cell morphology and distribution were imaged using confocal microscopy (Leica TCS SP8 X).

### Cell and ECM Characterization—Collagen Quantification

Samples taken from the large scaffolds were freeze‐dried and papain digested (Merck P3125). The digested samples were then freeze‐dried again and hydrolyzed with 0.4 м NaOH at 108 °C overnight. After hydrolysis, the samples were neutralized with 1.4 м citric acid. Chloramine‐T (Merck 2426) and incubated for 20 min at 170 rpm, followed by the addition of dimethylaminobenzaldehyde (Merck 3058). The reaction mixture was incubated at 60 °C for 20 min, and absorbance was measured at 570 nm using a microplate reader (Clariostar, Germany).

### Cell and ECM Characterization—DNA Quantification

DNA content was quantified using the Quant‐iT PicoGreen dsDNA Reagent Kit (ThermoFisher, P7589) following the manufacturer's instructions. Fluorescence was measured using a microplate reader (Clariostar) set to 485 nm excitation and 520 nm emission.

### Cell and ECM Characterization—GAG Quantification

Glycosaminoglycans were quantified by dimethylmethylene blue colorimetric assay (DMMB, Sigma–Aldrich, USA) following the manufacturer's instructions, and corrected for the DNA content. GAG content of the samples was measured after the reaction of the DMMB dye with a microplate reader (Clariostar), measuring the absorbance at 525 nm.

### Statistics

Statistical differences were determined using ANOVA with multiple comparisons, performed in Prism 10 (GraphPad Prism 10.4.0). The sample sizes were as follows: biochemistry (*n* = 6), printability and mechanical analysis (*n* = 5), TFM and condyle prints (*n* = 3). A difference was considered significant if *p* ≤ 0.05. The Alamar Blue assay was conducted on a single sample, precluding statistical analysis.

## Conflict of Interest

The authors declare no conflict of interest.

## Supporting information



Supporting Information

## Data Availability

The data that support the findings of this study are available from the corresponding author upon reasonable request.
