# Supplementary material for: Engineering Anisotropic Mechanical Properties in Large‐Scale Fabricated Cartilage Constructs Using Microfiber Reinforcement
Source: Adv Healthc Mater. 2025 Jun 8;14(19):2501014. doi: 10.1002/adhm.202501014 (PMC12304834; doi:10.1002/adhm.202501014)
Supplement: Supplementary file 1 — Supporting Information [file ADHM-14-0-s001.docx]

Supporting Information

**Figure S1:** Overview of the scaffold designs. A = Anisotropic scaffolds, different fiber spacing for local and surrounding fibers. H = Homogenous scaffolds, equal fiber spacing for local and surrounding fibers.

|  | | Surrounding fiber spacing | | | | 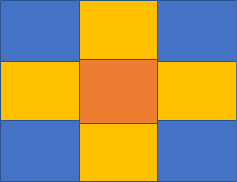 |
| --- | --- | --- | --- | --- | --- | --- |
|  |  | 200 | 300 | 400 | 500 |  |
| Local fiber spacing | 200 | H | A | A | A |  |
|  | 300 | A | H | A | A |  |
|  | 400 | A | A | H | A |  |
|  | 500 | A | A | A | H |  |

**Figure S2**: Custom casting mold for big anisotropic scaffolds


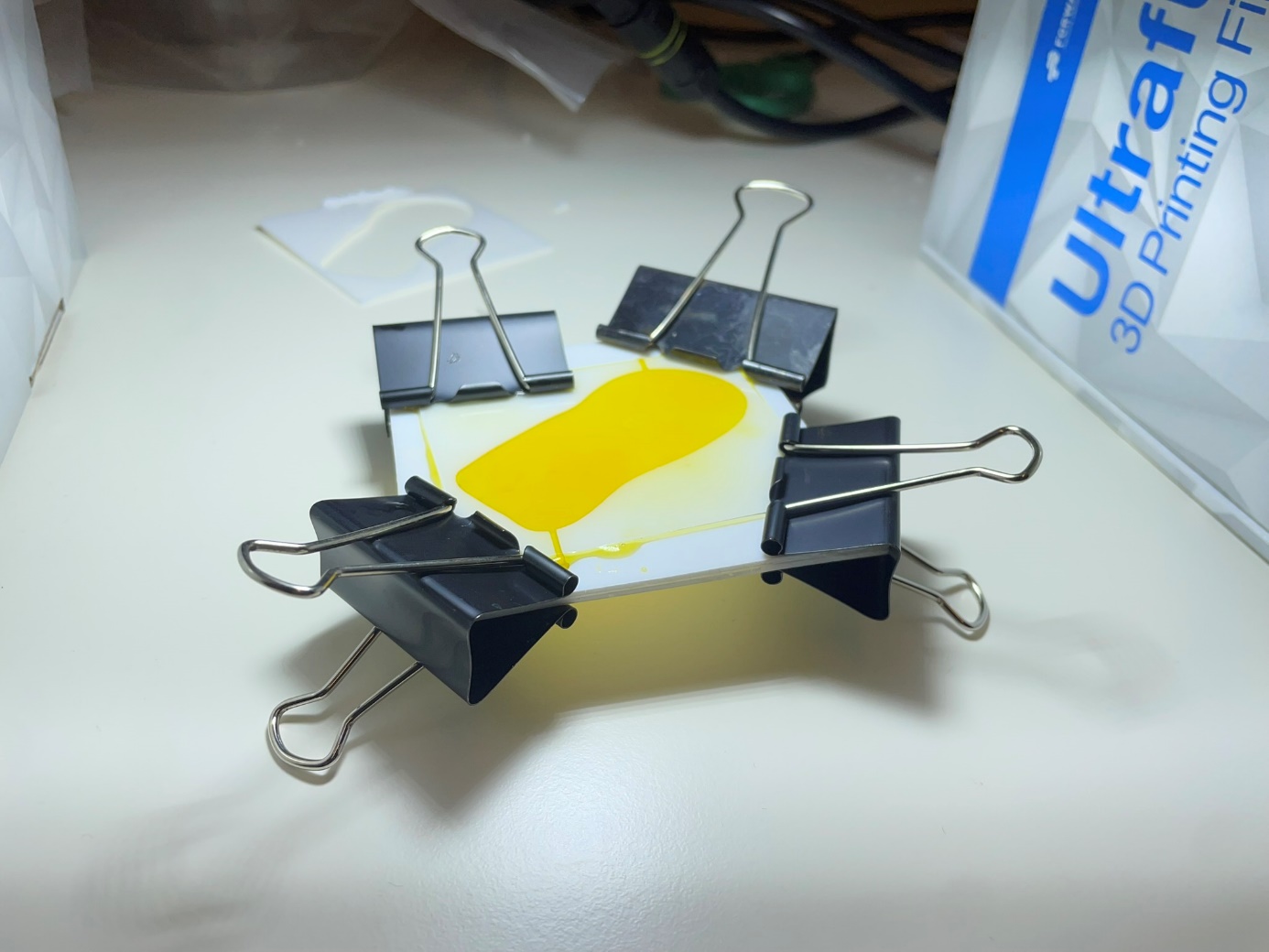


**Figure S3**: Measurement frame for large mechanical testing of large size anisotropic construct.


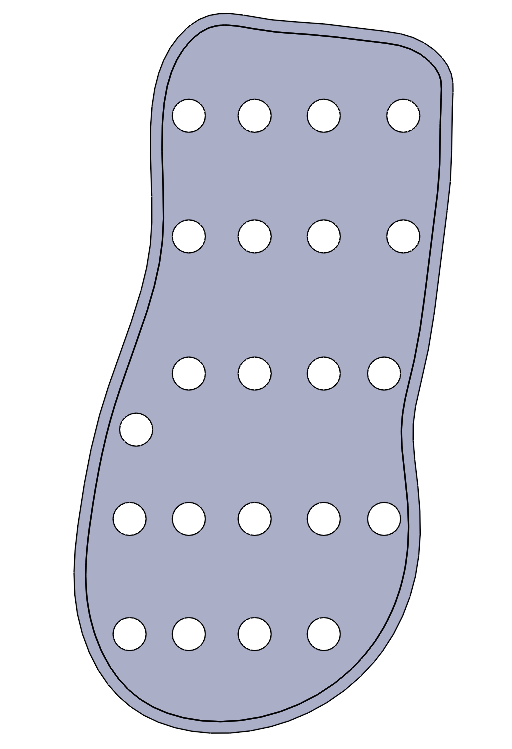


**Figure S4**: Relative printing error from zero.
